# Myeloid sarcoma arising at the uterine cervix in a patient with intestinal Behçet’s disease and concurrent myelodysplastic syndrome: A case report

**DOI:** 10.1097/MD.0000000000031559

**Published:** 2022-10-28

**Authors:** Hwa Yeon Choi, Min Gyoung Pak, Jung-Woo Park

**Affiliations:** a Department of Obstetrics and Gynecology, Dong-A University College of Medicine, Seo-gu, Busan, Republic of Korea; b Department of Pathology, Dong-A University College of Medicine, Seo-gu, Busan, Republic of Korea.

**Keywords:** case report, intestinal Behçet’s disease, myeloid sarcoma and myelodysplastic syndrome, uterine cervix

## Abstract

Myeloid sarcoma (MS) is an extramedullary tumor that consists of myeloblasts and rarely involves the female reproductive organs. Intestinal Behçet’s disease (BD) is a chronic, inflammatory illness that is often associated with myelodysplastic syndrome (MDS). When MDS is diagnosed, some patients with intestinal BD experience synchronous gastrointestinal flares.

**Case presentation:**

We report the case of a 49-year-old woman who presented with vaginal bleeding and an incidentally identified MS in the uterine cervix. Subsequent bone marrow biopsy showed simultaneous MDS without chromosomal abnormalities. This is the first reported case of concomitant MS, myelodysplastic disease, and intestinal BD.

**Conclusions:**

The accurate diagnosis of MSs that develop at non-predominant sites is crucial for a positive patient prognosis. MDS should be suspected in patients with a history of intestinal BD diagnosed with MS.

## 1. Introduction

Myeloid sarcoma (MS) is a rare malignant tumor consisting of myeloblasts.^[1]^ It is usually found at extramedullary sites but rarely manifests in the female reproductive organs. MS may simultaneously occur with acute myeloid leukemia (AML), myeloproliferative disorder, or myelodysplastic syndrome (MDS).^[1]^

Behçet’s disease (BD) is a chronic inflammatory disease of uncertain etiology, with gastrointestinal involvement reported in a quarter of the cases, typically in the ileocecal region. Intestinal BD has been associated with MDS, although the mechanisms underlying their relationship remain unclear.^[2,3]^

Here, we present a case of MS arising in the uterine cervix of a patient with intestinal BD. She was concomitantly diagnosed with MDS without chromosomal aberrations.

## 2. Case presentation

A 49-year-old nulliparous woman presented with vaginal bleeding for the last 3 weeks. Speculum examination revealed a spherical mass protruding through the external cervical os. Transvaginal ultrasonography revealed a 3.4-cm-sized submucosal fibroid mass below the uterine cervix. She had a history of intestinal BD with recently worsening symptoms and received 1500 mg of mesalazine twice a day and 5 mg of oral prednisolone once a day. She denied symptoms other than vaginal bleeding. Previous hematologic disorders had not been reported, and the patient’s surgical history was unremarkable. The initial complete blood count revealed normocytic anemia (hemoglobin 10.6 g/dL, hematocrit 31.9%, MCV 96.1/FL) and thrombocytopenia (130 × 10^3^/μL). The total white blood cell count was 5430/μL, without a differential count.

**Figure 1.**
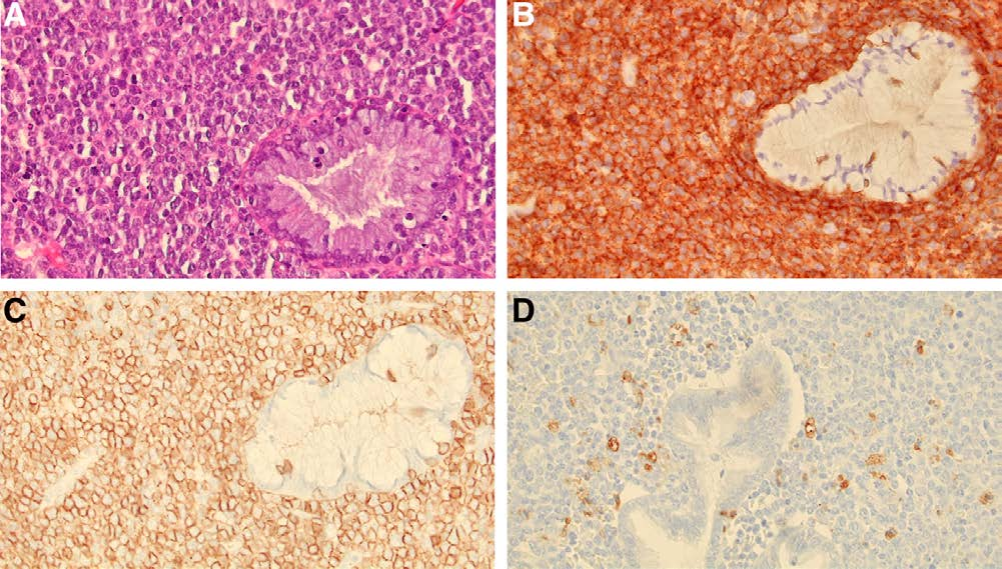
Pathologic findings. (A) Tumor cells effacing the normal architecture of the uterine cervix. The non-neoplastic endocervical gland maintains a normal structure (right). The tumor consists of blasts with scant cytoplasm and oval nuclei (hematoxylin-eosin stain, ×400 magnification). (B) Tumor cells exhibiting diffuse membranous positivity for leukocyte common antigen (LCA) immunohistochemical stain. A normal endocervical gland exhibiting negative immunostaining results (LCA, ×400 magnification). (C) Immature myeloid tumor cells that are positive for cluster of differentiation 56 (CD56) immunohistochemical stain (CD56, ×400 magnification). (D) Some tumor cells are positive for CD68 immunostaining, which is a marker associated with the myeloid lineage (CD68, ×400 magnification).

The patient underwent hysteroscopic mass resection. Histologically, the normal architecture of the uterine cervix was effaced by tumor cells, although some non-neoplastic endocervical glands maintained their normal structure. The tumor comprised relatively monomorphic cells with scant cytoplasm and hyperchromatic oval nuclei. The tumor cells exhibited diffuse membranous positivity for leukocyte common antigen and cluster of differentiation 56 (CD56) during immunohistochemical staining, whereas normal endocervical glands were negative for both markers. Some tumor cells were positive for CD68, a marker associated with the myeloid lineage, but were negative for myeloperoxidase (MPO) and terminal deoxynucleotidyl transferase. The final pathological diagnosis was MS (Fig. 1).

Her peripheral blood smear was performed after pathological diagnosis and revealed normocytic normochromic anemia (hemoglobin 8.7 g/dL) and thrombocytopenia (89 × 10^3^/μL). The white blood cell count was adequate (3330/μL), and the differential count revealed segmented neutrophils (59%), lymphocytes (34%), and monocytes (7%).

Subsequent bone marrow aspiration and biopsy revealed approximately 10% hypocellular bone marrow with 2.2% blasts, dysgranulopoiesis, and myelofibrosis. Cytogenetic analysis revealed a normal karyotype, and next-generation sequencing and fluorescence in situ hybridization revealed negativity for genetic mutations of AML. The diagnosis was unclassifiable MDS with MS of the uterine cervix.

## 3. Discussion and conclusions

MS is often associated with AML and occasionally coincides with MDS.^[4,5]^ The progression rate of MDS to AML is 5% at 2 years post-MDS diagnosis and 15% at 5 years post-MDS diagnosis.^[6]^ MS occurring with MDS is considered a poor prognostic factor due to the impending transformation to AML.^[7]^

Despite its clinical significance, the histological diagnosis of MS in atypical locations is difficult. The predominant sites of MS are the skin, bone, and lymph nodes; the female reproductive organs are rarely involved.^[8]^ Due to its rarity, few studies have assessed the sites of MS in the female reproductive system, with inconsistent results. Garcia et al^[9]^ reported that the uterus, including the cervix, is the most frequently affected location of the reproductive system in MS. Conversely, another study identified the ovary as the most commonly affected site, followed by the vagina.^[10]^ As MS can occur anywhere other than the bone marrow, its clinical manifestations vary widely and rely on the tumor location. Four-fifths of patients with MS arising in the uterine cervix experience vaginal bleeding.^[11]^

The immunohistochemical analysis could be established as a standard for the accurate diagnosis of MS. Pileri et al^[7]^ reported CD68 as the most frequently expressed marker of MS. Additional markers, such as MPO, CD117, CD99. and CD56, may also aid in MS detection.^[7]^ CD56 has been implicated in extramedullary involvement, particularly in the skin, and has been associated with a high incidence of MS.^[7]^ Although several studies have shown that all MS that develop in the gynecologic tract are positive for MPO,^[8,10]^ MPO may also be diversely expressed in MS originating from the female reproductive organs.

Kawamoto et al^[12]^ analyzed the karyotype of 56 patients with MS to investigate specific genetic variations. They reported various chromosomal aberrations but an absence of specific mutations correlating with MS. This suggests that genetic alterations and MS development may not be directly related.

In our case, the patient had intestinal BD as the underlying disease. Esatoglu et al^[3]^ reported that 3 of 7 patients with intestinal BD preceding MDS experienced aggravating gastrointestinal symptoms when concomitantly diagnosed with MDS. The worsening course of intestinal BD may be indicative of MDS development. The clinical manifestations of MDS are often nonspecific, asymptomatic, and include an abnormal complete blood count. Bleeding is a common symptom of thrombocytopenia; however, close observation is required when patients with intestinal BD present with cytopenia during follow-up.

This is the first case in which a patient with intestinal BD was simultaneously diagnosed with MS and MDS. When MS is detected in the uterine cervix, correct diagnosis, supported by immunohistochemistry and identification of bone marrow involvement, is crucial for successful intervention. In particular, MDS should be suspected when MS is detected in cases of severe intestinal BD.

## Author contributions

**Conceptualization:** Hwa Yeon Choi, Jung-Woo Park.

Hwa Yeon Choi.

**Investigation:** Hwa Yeon Choi.

**Methodology:** Hwa Yeon Choi, Jung-Woo Park.

**Supervision:** Jung-Woo Park.

**Visualization:** Min Gyoung Pak.

**Writing—original draft:** Hwa Yeon Choi, Min Gyoung Pak

**Writing—review and editing:** Min Gyoung Pak, Jung-Woo Park
